# Identification of biomarkers associated with the invasion of nonfunctional pituitary neuroendocrine tumors based on the immune microenvironment

**DOI:** 10.3389/fendo.2023.1131693

**Published:** 2023-07-14

**Authors:** Jiangping Wu, Jing Guo, Qiuyue Fang, Yulou Liu, Chuzhong Li, Weiyan Xie, Yazhuo Zhang

**Affiliations:** ^1^Beijing Neurosurgical Institute, Capital Medical University, Beijing, China; ^2^Department of Neurosurgery, Beijing Tongren Hospital Affiliated to Capital Medical University, Beijing, China; ^3^Department of Neurosurgery, Beijing Tiantan Hospital Affiliated to Capital Medical University, Beijing, China; ^4^Center of Brain Tumor, Beijing Institute for Brain Disorders, Beijing, China; ^5^China National Clinical Research Center for Neurological Diseases, Beijing, China

**Keywords:** nonfunctioning pituitary neuroendocrine tumors (NF-PitNEts), invasive, immune microenvironment, WGCNA, biomarkers

## Abstract

**Introduction:**

The invasive behavior of nonfunctioning pituitary neuroendocrine tumors (NF-PitNEts) affects complete resection and indicates a poor prognosis. Cancer immunotherapy has been experimentally used for the treatment of many tumors, including pituitary tumors. The current study aimed to screen the key immune-related genes in NF-PitNEts with invasion.

**Methods:**

We used two cohorts to explore novel biomarkers in NF-PitNEts. The immune infiltration-associated differentially expressed genes (DEGs) were obtained based on high/low immune scores, which were calculated through the ESTIMATE algorithm. The abundance of immune cells was predicted using the ImmuCellAI database. WGCNA was used to construct a coexpression network of immune cell-related genes. Random forest analysis was used to select the candidate genes associated with invasion. The expression of key genes was verified in external validation set using quantitative real-time polymerase chain reaction (qRT‒PCR).

**Results:**

The immune and invasion related DEGs was obtained based on the first dataset of NF-PitNEts (n=112). The immune cell-associated modules in NF-PitNEts were calculate by WGCNA. Random forest analysis was performed on 81 common genes intersected by immune-related genes, invasion-related genes, and module genes. Then, 20 of these genes with the highest RF score were selected to construct the invasion and immune-associated classification model. We found that this model had high prediction accuracy for tumor invasion, which had the largest area under the receiver operating characteristic curve (AUC) value in the training dataset from the first dataset (n=78), the self-test dataset from the first dataset (n=34), and the independent test dataset (n=73) (AUC=0.732/0.653/0.619). Functional enrichment analysis revealed that 8 out of the 20 genes were enriched in multiple signaling pathways. Subsequently, the 8-gene (BMP6, CIB2, FABP5, HOMER2, MAML3, NIN, PRKG2 and SIDT2) classification model was constructed and showed good efficiency in the first dataset (AUC=0.671). In addition, the expression levels of these 8 genes were verified by qRT‒PCR.

**Conclusion:**

We identified eight key genes associated with invasion and immunity in NF-PitNEts that may play a fundamental role in invasive progression and may provide novel potential immunotherapy targets for NF-PitNEts.

## Introduction

Pituitary neuroendocrine tumors (PitNEts) account for approximately 10-20% of intracranial tumors and are the second most common neoplasms of the central nervous system (1, 2). The prevalence of PitNEts ranges from 76-116 cases per 100,000 population, and the incidence is between 3.9 and 7.4 cases per 100,000 per year (3). These tumors are classified into functional and nonfunctioning pituitary tumor subtypes according to endocrine status (4). Nonfunctional pituitary neuroendocrine tumors (NF-PitNEts) account for 36%-54% of PitNEts and are usually detected based on signs and symptoms (headache, visual disturbance, and/or hypopituitarism) related to the effects of tumor mass because of the lack of excessive hormone secretion (2, 4–6). In this context, surgery is the treatment of choice because it can rapidly achieve decompression and symptomatic improvement (7–9). Because most macro-NF-PitNEts have the potential to invade the surrounding structures, such as the cavernous sinus or the sphenoid sinus, complete resection is often challenging and is achieved in up to 60-73% of patients (10, 11). Moreover, invasive tumors have an increased recurrence rate due to tumor residues, which require additional surgery or radiation therapy and thus pose a further risk of complications (12–14). As a result, it is necessary to explore the pathogenesis of invasive NF-PitNEts to optimize the treatment of this tumor.

The tumor immune microenvironment (TIME) plays a crucial role in tumor development, progression, and immunotherapy (15, 16). The TIME is composed of immune cells (lymphocytes and macrophages), immune-related pathways and cytokines secreted by tumor cells or immune cells (17). Pituitary tumor cells have been shown to recruit a variety of tumor-infiltrating immune cells, such as macrophages, T lymphocytes, B lymphocytes, FOXP3+ cells, neutrophils, and NK cells, into the tumor microenvironment (18–20). Moreover, the TIME has many effector functions and may promote the proliferation, migration and invasion of pituitary tumors (18, 21, 22). Therefore, it is essential to comprehensively analyze immunological genes affecting the abundance of immune cells in the invasive NF-PitNEts microenvironment.

In the current study, differentially expressed genes (DEGs) were identified at the tumor invasive and immune levels. Weighted correlation network analysis (WGCNA) was used to screen immune cell-related genes. The key invasive and immunological genes were further investigated by constructing a classification model and enrichment analysis. Our study screened out critical invasive-immune associated genes, which could provide new ideas for exploring immunological studies and some potential treatment strategies for NF-PitNEts patients.

## Materials and methods

### Human tissue samples and clinical data

In this study, we used two cohorts to explore novel biomarkers in pituitary tumors. The first dataset included 112 patients, and another independent test dataset contained 73 patients. Tumor specimens were obtained from patients with NF-PitNEts (n=112) who underwent transsphenoidal surgical resection at Beijing Tiantan Hospital between June 2018 and July 2019. The diagnosis of NF-PitNEts is defined as the absence of clinical and biochemical evidence of overproduction of adenohypophysis hormone. The mean age of these 112 patients was 52 years (range, 21-75), and there were 63 males and 49 females. The demographics and clinicopathological features of the patients are summarized in **Table 1**. Tumor cavernous sinus (CS) invasion was defined as Knosp grade 3 and 4 or intraoperative evidence (23). The expression profiles and matching clinical information of independent test datasets (n=73) were described previously (24). In addition, 16 NF-PitNEts specimens (8 invasive and 8 noninvasive) were collected from the same hospital as an independent validation cohort (**Table S3**), and their expression levels were verified by quantitative real-time polymerase chain reaction (qRT-PCR). This study recruitment process and protocol were approved by the Medical Ethics Committee of Beijing Tiantan Hospital, and informed consent was obtained from all individual participants.

**Table 1:** Clinical information of 112 NF-PitNEts patients.

Variables	Group	N (%)
Age	≤52	57 (51%)
	>52	55 (49%)
Gender	Male	63 (56%)
	Female	49 (44%)
Tumor size classification	Macro	20 (18%)
	Giant	92 (82%)
CS Invasion	Yes	51 (46%)
	No	61 (54%)
Histological types	GTs	75 (67%)
	SCTs	34 (30%)
	NCTs	3 (3%)

CS, cavernous sinus; GTs, gonadotroph tumors; SCTs, silent corticotroph tumors; NCAs, null cell tumors.

### Total RNA extraction and RNA sequencing

A total of 1-3 μg RNA per sample was extracted and purified from the collected specimens of NF-PitNEts. According to the instructions provided, sequencing libraries were constructed using the NEBNext^®^ Ultra™ RNA Library Prep Kit for Illumina^®^ (#E7530L, NEB, USA). After the library was successfully generated (effective concentration >10 nM), the index-coded samples were clustered on the cBot cluster generation system using HiSeq PE Cluster Kit v4-cBot-HS (Illumina). The library was then sequenced on an Illumina platform, and 150-bp paired-end reads were generated. Raw data were filtered with FAST-QC, and the clean reads were then mapped to the human genome hg19 sequence (GRCh37) using HISAT2 (25). HTseq was used to generate gene counts, and the RPKM method was used to determine gene expression (26).

### Differential expression analysis

The Estimation of Stromal and Immune cells in Malignant Tumor tissues using Expression data (ESTIMATE) algorithm (27) was used to obtain the immune levels of 112 pituitary tumor patients. Based on the median immune score, patients were divided into high and low groups. These 112 patients were also divided into invasion and noninvasion groups based on their clinical invasion information.

The “limma” R package was used to obtain the DEGs from the high vs. low immune score groups and invasion vs. noninvasion groups. Genes with an adjusted P value < 0.05 and |log2-fold change| ≥ 0.585 were filtered as DEGs.

### Weighted gene coexpression network analysis

First, the ImmuCellAI database was applied to estimate the abundance of 24 immune cell types in the 112 patients with NF-PitNEts (28). The abundance of these immune cells in the invasion and noninvasion groups was then used for WGCNA.

Second, the “WGCNA” R package was applied to build a coexpression network of immune cell-related genes. Next, several gene modules were detected based on their similar expression patterns. Finally, the abundance of immune cells was associated with these gene expression modules, and genes in the modules were selected for further analysis.

### Random forest analysis

The “randomForest” R package was used to select the candidate genes associated with invasion. We first divided the 112 patients into a training dataset (n=78) and a self-test (n=34) dataset based on the “sample” function of the R program. Then, random forest analysis (RF) was performed through the “randomForest” function in the training dataset, the error rate curve was drawn and changes in the error rate of different numbers of genes selected were observed. Finally, the “ggplot” R package was used to show the MeanDecreaseAccuracy and the best RF model of these genes.

Genes with the top 20 MeanDecreaseAccuracy were used to build the SVM model through the “e1071” R package, and the “pROC” package was used to perform the classification efficiency of the model in the training dataset, self-test dataset and independent test dataset.

### Consensus clustering

The R package “ConsensusClusterPlus” was applied to explore the classification efficiency of 8 crucial genes. Subsequently, the “pROC” package was used to show the classification efficiency of these genes.

### Enrichment analysis

Functional enrichment analysis was carried out by the “clusterProfiler” R package, and only functions with a p value<0.05 were selected.

### qRT-PCR assay

The QIAGEN RNeasy Kit and High Capacity cDNA Reverse Transcription Kit were used to extract total RNA from the verified samples and conduct reverse transcription reactions. qRT-PCR was performed in a volume of 20 µl with Power SYBR™ Green PCR Master Mix on a QuantStudio 3 and 5 System (Applied Biosystems). GAPDH was used as a housekeeping control. The sequences of the primers are shown in **Table 2**.

**Table 2:** Primers used for qRT-PCR.

Gene	Forward primer (5′-3′)	Reverse primer (3′-5′)
BMP6	CCTTACGACAAGCAGCCCTT	TGGGACTGGGTAGAGCGATT
CIB2	GCGTTTTCCGAGGATGGTGA	CCTTGCAGATGAAGTTGTCAGTG
FABP5	GGAAGGAAAGCACAATAACAA	TTCATAGATCCGAGTACAGG
HOMER2	ACCTGGAAGACAAAGTGCGT	TGCAGGTCGTCAATCTTCCC
MAML3	GTTTCAAGGTTCTCCCCAGGAT	ATTCCCATCATGCCTGCGTT
NIN	AAGTTTGGTGACCTCGATCCT	TGGTCTTGTAGTACCCTGCAC
PRKG2	ACACGACGACCTGAGGATTT	GTGCTTTCAGTCCCTCCCAA
SIDT2	ATGAGTTCCCTGAAGGCGTG	AGGCTACGTTGTTGTCCAGG
GAPDH	GCCATCACTGCCACTCAGAAGA	ATGACCTTGCCCACAGCCTTG

### Statistical analysis

All statistical analyses were performed by R software (version 3.6.3). The bar plot between two different groups was drawn by the “ggplot” package, and a T test was used to compare the differences. A P value <0.05 was considered significant.

## Results

### Identification of immune infiltration-associated and invasion-associated DEGs in pituitary tumor patients

**Figure 1:**
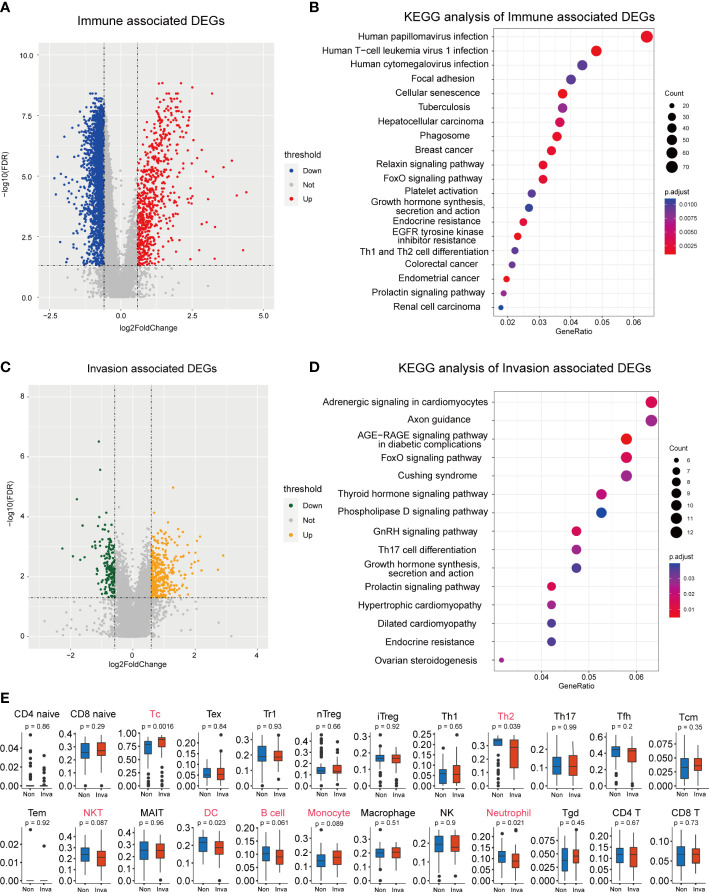
Screening for immune and invasion-related genes in pituitary tumors. **(A)** Volcano plot showing the differentially expressed genes (DEGs) between high Immune-score samples and low Immune-score samples. **(B)** The functional enrichment analysis of the Immune-related DEGs. **(C)** Volcano plot showing the differentially expressed genes (DEGs) between patients with and without invasion. **(D)** The functional enrichment analysis of the Invasion-related DEGs. **(E)** Multiple immune cell differences between patients with and without invasion.

ESTIMATE analysis was performed in the 112 NF-PitNEts expression data, and the samples were divided into high- and low-immune score groups. Next, 3152 DEGs were obtained from the high- vs. low-immune score groups, including 662 upregulated and 2490 downregulated genes (**Figure 1A** and **Table S1**). The enrichment analysis indicated that these DEGs were involved in Th1 and Th2 cell differentiation, the relaxin signaling pathway, and the FoxO signaling pathway (**Figure 1B**). Then, we obtained 525 DEGs (352 upregulated and 173 downregulated) associated with invasion (**Figure 1C** and **Table S2**). These genes are related to the FoxO signaling pathway and Th17 cell differentiation (**Figure 1D**). This finding reveals that invasion-associated DEGs are involved in the immune-related functions of pituitary tumors.

Subsequently, we predicted the immune cell abundance of these patients. Cytotoxic T cells (Tc), Th2 cells, natural killer T cells (NKT), dendritic cells (DC), B cells, monocytes and neutrophils differed between the invasion and noninvasion groups (**Figure 1E**).

### Immune cell-associated modules in pituitary tumors

**Figure 2:**
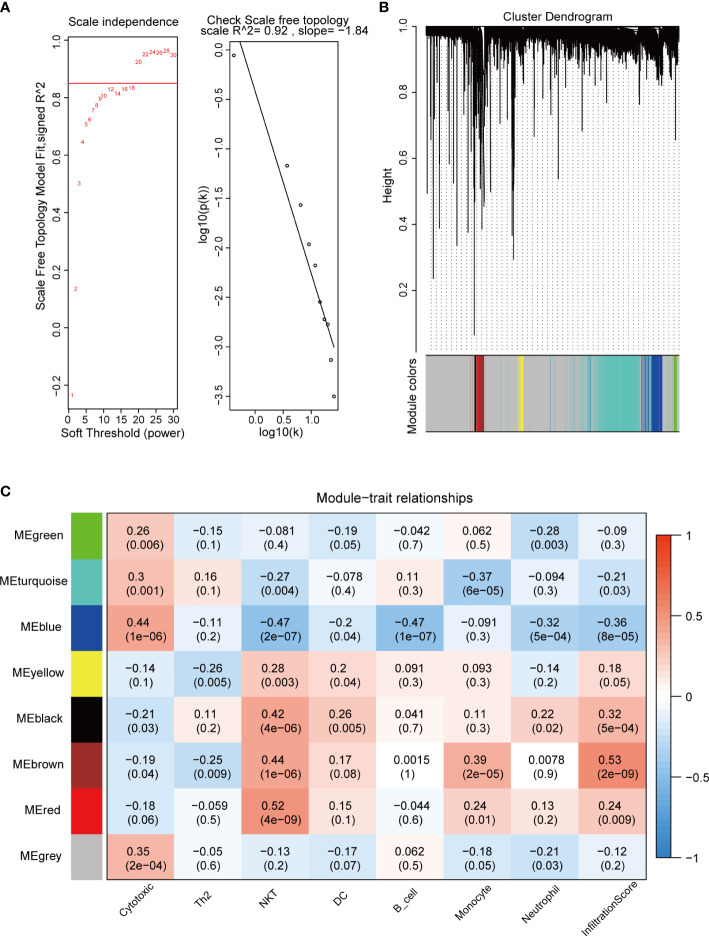
WGCNA of crucial immune cells in pituitary tumors. **(A)** Soft power selection of the WGCNA network. Here, we selected 20 as the power. **(B)** Clustering dendrogram of genes with dissimilarity based on topological overlap and assigned module colors. **(C)** The relationships between gene modules and immune cells. The P value is shown in parentheses.

We used the “WGCNA” package to calculate the immune cell-associated modules in pituitary tumors. The soft power of the coexpression network was calculated through the “*pickSoftThreshold*” function and was set to 20 (**Figure 2A**). At this power, the R^2^ of the scale-free topology model under the soft threshold was 0.92, which indicates that the network conformed to the scale-free feature (**Figure 2A**). Then, the network was constructed, and seven coexpression modules (green, turquoise, blue, yellow, black, brown, and red) were built (**Figure 2B**). Subsequently, the correlation between these seven modules and immune cell abundance was calculated. The green module was significantly correlated with neutrophils; the turquoise module was significantly correlated with monocytes; the blue module was significantly correlated with cytotoxic cells; and the yellow, black, brown, and red modules were significantly associated with NKT cells (**Figure 2C**). These results suggest that these genes are immune-related in the tumor microenvironment of pituitary tumors.

### Construction of the invasion and immune-associated classification model (IICM)

**Figure 3:**
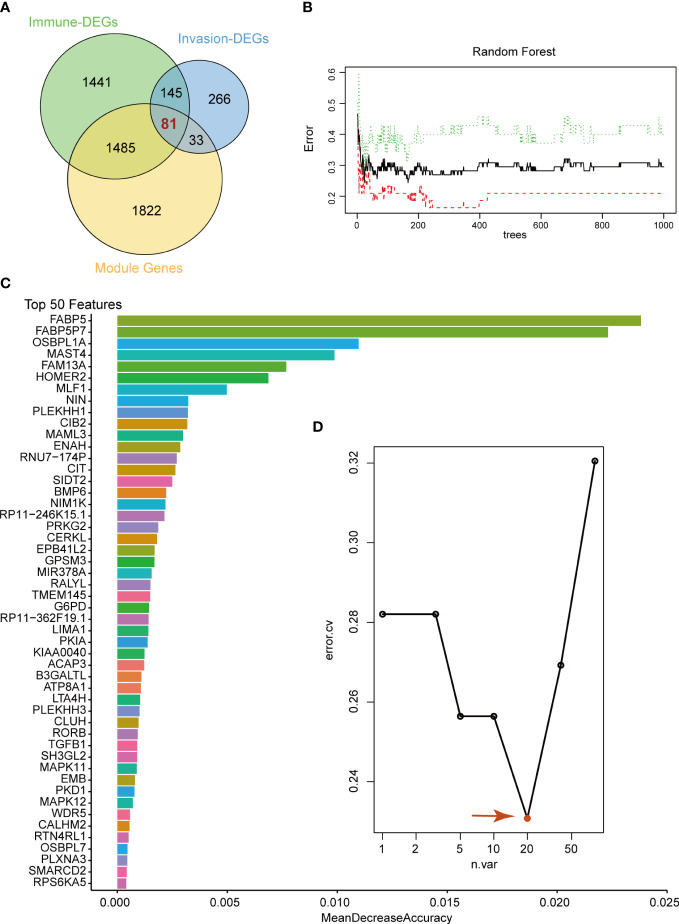
Random Forest analysis. **(A)** Venn diagram showing the candidate genes between immune-related DEGs, invasion-related DEGs and WGCNA modules. We selected 81 common genes for random forest analysis. **(B)** Random Forest analysis of the 81 genes. **(C)** Top 50 genes with the lowest MeanDecreaseAccuracy in random forest analysis. **(D)** The lowest error rate model contains 20 candidate genes based on random forest analysis.

To select the candidate crucial genes in pituitary tumors, we first set the intersection of immune-related DEGs, invasion-related DEGs and module genes and found 81 common genes (**Figure 3A**). Next, we used the 81 common genes to perform RF analysis. We started the RF analysis by generating 1000 decision trees, which showed a lower error rate (**Figure 3B**). Then, the random forest results indicated that, when the number of candidate genes was 20, the classification efficiency error rate of the model was the lowest. We showed the top 50 accuracies of these genes and selected the top 20 for further analysis (**Figures 3C, D****).**

**Figure 4:**
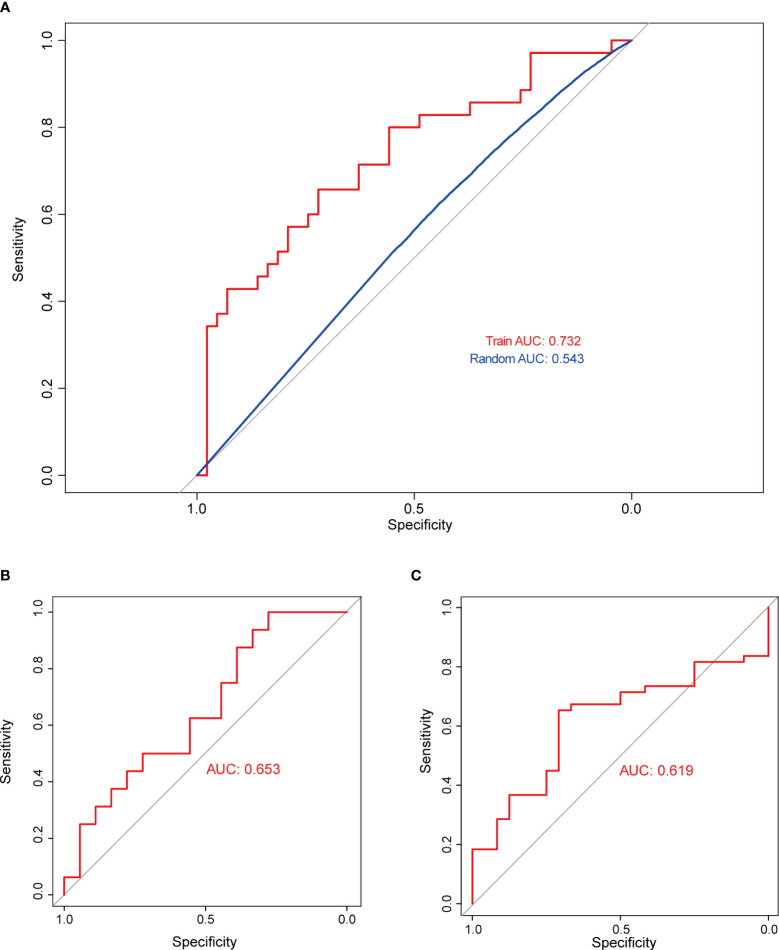
Validation of model classification performance. **(A)** The ROC curve of the RF model with 20 genes in the training dataset; the AUC reached 0.732. **(B)** The RF model’s classification performance in the self-test dataset; the AUC reached 0.653. **(C)** The RF model’s classification performance in the independent test dataset; the AUC reached 0.619.

Subsequently, SVM analysis was used to construct the IICM (**Figure 4A**). We found that this model could distinguish the invasive and noninvasive patients in the training dataset (AUC=0.732, **Figure 4A**). It also exhibited good efficacy in the self-test dataset (AUC=0.653, **Figure 4B**) and independent test dataset (AUC=0.619, **Figure 4C****).** The results showed that this model could be used to predict the invasion state of pituitary tumors.

### Eight genes in IICM exhibit better efficacy in pituitary tumors

**Figure 5:**
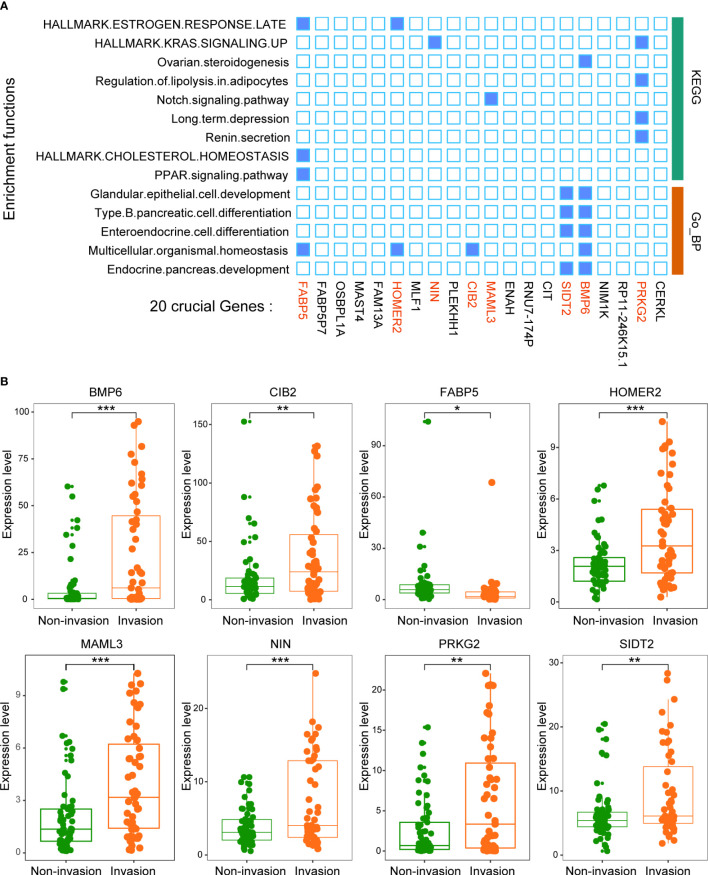
Twenty crucial genes were enriched in multiple pathways. **(A)** Functional enrichment analysis of the 20 genes. The results showed that 8 genes played an important role in these pathways. **(B)** The expression levels of the 8 crucial genes between patients with and without invasion. *p < 0.05, **p < 0.01, ***p < 0.001.

We then analyzed the function of these 20 key genes. The results suggested that 8 of the 20 genes were enriched in multiple signaling pathways, such as the KRAS signaling pathway and PPAR signaling pathway (**Figure 5A**). This result suggests that the function of this model is mainly driven by these 8 genes (BMP6, CIB2, FABP5, HOMER2, MAML3, NIN, PRKG2 and SIDT2).

**Figure 6:**
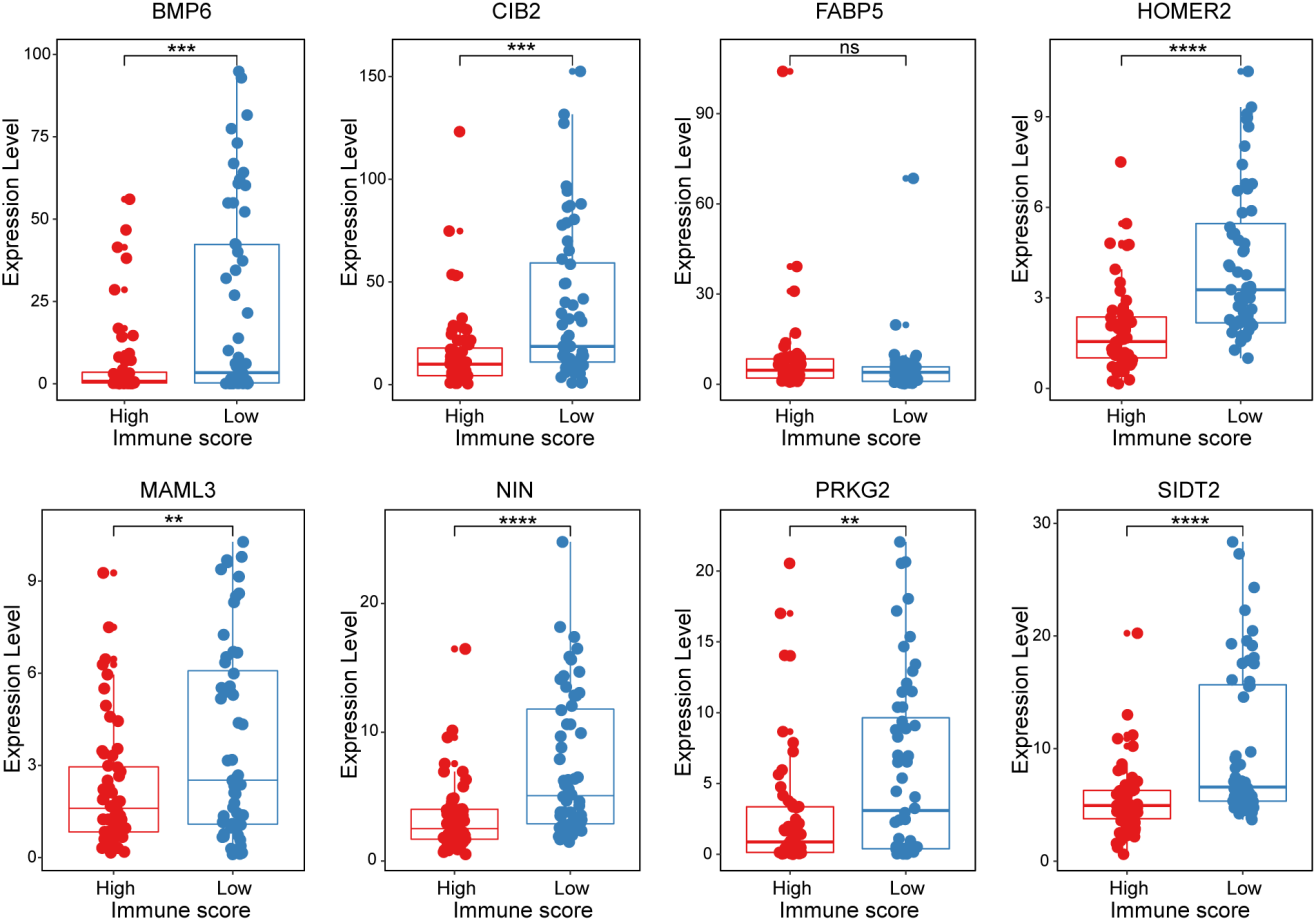
The expression levels of the 8 crucial genes in high Immune-score samples and low Immune-score samples. The results are expressed as the means ± SD (Student’s t test. **p < 0.01, ***p < 0.001, ****p < 0.0001). ns, no significance.

**Figure 7:**
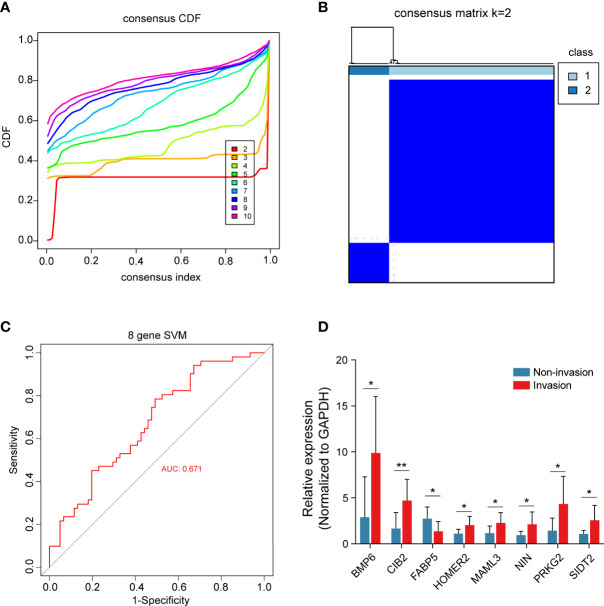
Consensus cluster of the 8 crucial genes. **(A)** Consensus index of the consensus cluster analysis. The 8 crucial genes could divide the patients into two groups. **(B)** Heatmap of the two groups divided by the 8 genes. **(C)** The new model constructed by the 8 crucial genes performed with good classification effectiveness in pituitary tumors. **(D)** Validation of the 8 crucial genes between 8 CS invasive NF-PitNEts and 8 noninvasive NF-PitNEts. The results are expressed as the means ± SD (Student’s t test. *p < 0.05; **p < 0.01).

All 8 genes were differentially expressed in the invasion and noninvasion groups (**Figure 5B**), and most were differentially expressed in the high- and low-immune score groups (**Figure 6**). Subsequently, the consensus clustering analysis suggested that the 8 genes could well divide the patients into two groups (**Figures 7A, B**). We then constructed a classifier for these 8 genes, and the results showed that the 8-gene classification model showed good classification efficiency in pituitary tumors (AUC=0.671, **Figure 7C**). Then, qRT-PCR was performed to validate the expression level of these genes between invasive and noninvasive NF-PitNEts (**Figure 7D**). Consistent with the sequencing data, the mRNA expression of BMP6, CIB2, HOMER2, MAML3, NIN, PRKG2, and SIDT2 was significantly upregulated in invasive NF-PitNEts, while FABP5 was significantly downregulated. Overall, we filtered 8 genes that could predict the invasion status of pituitary tumors and could be used as predictors for further treatment of the tumors.

## Discussion

Although NF-PitNEts are benign neoplasms, they often invade surrounding structures and cannot be cured using standard therapies (29). Moreover, invasion is known as an important prognostic factor for recurrence (30). Recent studies have reported that immune cells infiltrate pituitary adenomas and may play an important role in tumor invasion and progression (31–34). Therefore, understanding the mechanisms involved in immunity with invasive NF-PitNEts could lead to the discovery of new therapeutic targets in the future.

First, 3152 DEGs (662 upregulated and 2490 downregulated) were identified based on the high- and low-immune score groups, which were obtained using the ESTIMATE algorithm. We obtained 525 DEGs between invasive and noninvasive NF-PitNEts. Then, the abundance of immune cells was predicted using the ImmuCellAI database, and Tc, Th2, NKT, DC, B cell, monocyte and neutrophil cells were found to be different between the invasion and noninvasion groups. Huang X et al. (35) found that patients with invasive NF-PitNEts had significantly lower CD3-CD56+ natural killer (NK) cells than patients with noninvasive NF-PitNEts in peripheral blood.

Subsequently, the abundance of these immune cells was used to construct a coexpression network of immune cell-related genes by WGCNA. As a bioinformatics method, WGCNA clustering results (coexpression gene modules) have high biological significance and reliability (36, 37). Next, the 81 interacting genes were identified by immune-related DEGs, invasion-related DEGs and module genes significantly associated with immune cells. To narrow down the number of invasion- and immune-associated genes, random forest analysis was performed. The 20 genes with the highest RF score were selected to constrict the SVM model, and the classification efficiency was verified in the training, self-test, and independent test datasets. This result indicated that these genes play an important role in invasive behavior. Then, functional enrichment analysis was performed on these 20 genes, and 8 were found to be enriched in multiple signaling pathways, such as the KRAS signaling pathway, Notch signaling pathway, and PPAR signaling pathway. Liu et al. (38) found that upregulation of secreted phosphoprotein 1 affects tumor cell proliferation, migration, and invasion *via* the KRAS/MEK pathway in head and neck cancer. Feng et al. (39) reported that the Notch signaling pathway was associated with the invasion of growth hormone adenomas. Finally, these 8 invasion- and immune-related genes (BMP6, CIB2, FABP5, HOMER2, MAML3, NIN, PRKG2 and SIDT2) were validated by consensus clustering analysis and verified by qRT-PCR between invasive and noninvasive NF-PitNEts.

Bone morphogenetic protein-6 (BMP-6) belongs to the TGF-β superfamily (40). BMP-6 was deemed to be associated with several tumor metastases, such as breast, prostate, rectal, and thyroid carcinomas (41–45). In addition, BMP-6 changes the morphology of macrophages and induces the expression of the cytokine tumor necrosis factor (TNF)-α (46). Calcium and integrin-binding protein 2 (CIB2) is a small EF-hand protein that can bind Mg^2+^ and Ca^2+^ ions and participates in basic cellular functions (47). Zhu et al. (48) found that CIB2 is correlated with cell proliferation, migration, and invasion in ovarian cancer. Moreover, Wang et al. (49) reported that CIB2 can cause M2 macrophage death and facilitate tumor microenvironment inflammation. Fatty acid-binding protein 5 (FABP5), which is an intracellular lipid carrier, is correlated with tumor development in multiple human cancers (50–52), including prostate cancer (53), bladder cancer (54), and glioblastoma (55). Liu et al. (56) reported that FABP5 promotes lipid accumulation in monocytes/macrophages and may represent a therapeutic target for tumor-associated monocytes (TAMs) and cancer cells. Homer scaffolding protein 2 (HOMER2) is an adaptor protein that has been reported to be associated with tumor progression in endometrial cancer (57). Mastermind-like 3 (MAML3) is a known transcriptional coactivator of NOTCH (58). Onishi et al. (59) found that the inhibition of MAML3 significantly reduced the proliferation and invasion of tumor cells in small cell lung cancer. SID1 transmembrane family member 2 (SIDT2) is a lysosomal membrane protein that promotes RNA degradation by transporting RNA to lysosomes (60, 61). Yi et al. (62) found that the expression of SIDT2 was associated with the biological behaviors of cancer cells in papillary thyroid carcinoma.

There are still some concepts that could improve our research. First, although we validated the IICM using a self-test dataset and another independent NF-PitNEts cohort, a large-scale multicenter cohort is needed for stronger validation. Second, our study only verified expression levels in NF-PitNEts tissues by qRT-PCR, but the underlying functions and mechanism of these genes still need to be explored *in vivo* and *in vitro*.

In summary, we conducted a comprehensive bioinformatic analysis and screened out immune-related genes that were significantly correlated with invasion in patients with NF-PitNEts. The current study may provide a novel potential immunotherapy target for invasive NF-PitNEts.

## Data availability statement

The data provided in the study are deposited in the Gene Expression Omnibus (https://www.ncbi.nlm.nih.gov/geo/), the accession number is: GSE169498, and another set of data is included in the supplementary material, further inquiries can be directed to the corresponding author/s.

## Ethics statement

The studies involving human participants were reviewed and approved by the Medical Ethics Committee of Beijing Tiantan Hospital. The patients/participants provided their written informed consent to participate in this study.

## Author contributions

WX, YZ worked on the conception and designed research, and eventually approved the manuscript. QF and YL was contributed to collected and analyzed clinical data of patients. JW and JG were dedicated to data analysis, interpretation, and drafting. All authors read and approved the final manuscript.

## References

[1] Ostrom QT Cioffi G Waite K Kruchko C Barnholtz-Sloan JS. CBTRUS statistical report: primary brain and other central nervous system tumors diagnosed in the united states in 2014-2018. Neuro Oncol (2021) 23:iii1–iii105. doi: 10.1093/neuonc/noab200 34608945 PMC8491279

[2] Herrgott GA Asmaro KP Wells M Sabedot TS Malta TM Mosella MS. Detection of tumor-specific DNA methylation markers in the blood of patients with pituitary neuroendocrine tumors. Neuro Oncol (2022) 24:1126–39. doi: 10.1093/neuonc/noac050 PMC9248407 35212383

[3] Daly AF Beckers A. The epidemiology of pituitary adenomas. Endocrinol Metab Clin North Am (2020) 49:347–55. doi: 10.1016/j.ecl.2020.04.002 32741475

[4] Molitch ME. Diagnosis and treatment of pituitary adenomas: a review. JAMA (2017) 317:516–24. doi: 10.1001/jama.2016.19699 28170483

[5] Sam AH Shah S Saleh K Joshi J Roncaroli F Robinson S. Clinical outcomes in patients with nonfunctioning pituitary adenomas managed conservatively. Clin Endocrinol (Oxf) (2015) 83:861–5. doi: 10.1111/cen.12860 26201671

[6] Ntali G Wass JA. Epidemiology, clinical presentation and diagnosis of non-functioning pituitary adenomas. Pituitary (2018) 21:111–8. doi: 10.1007/s11102-018-0869-3 29368293

[7] Greenman Y Tordjman K Kisch E Razon N Ouaknine G Stern N. Relative sparing of anterior pituitary function in patients with growth hormone-secreting macroadenomas: comparison with nonfunctioning macroadenomas. J Clin Endocrinol Metab (1995) 80:1577–83. doi: 10.1210/jcem.80.5.7745003 7745003

[8] Losa M Mortini P Barzaghi R Ribotto P Terreni MR Marzoli SB. Early results of surgery in patients with nonfunctioning pituitary adenoma and analysis of the risk of tumor recurrence. J Neurosurg (2008) 108:525–32. doi: 10.3171/JNS/2008/108/3/0525 18312100

[9] Nomikos P Ladar C Fahlbusch R Buchfelder M. Impact of primary surgery on pituitary function in patients with non-functioning pituitary adenomas – a study on 721 patients. Acta Neurochir (Wien) (2004) 146:27–35. doi: 10.1007/s00701-003-0174-3 14740262

[10] Penn DL Burke WT Laws ER. Management of non-functioning pituitary adenomas: surgery. Pituitary (2018) 21:145–53. doi: 10.1007/s11102-017-0854-2 29280026

[11] Hansen TM Batra S Lim M Gallia GL Burger PC Salvatori R. Invasive adenoma and pituitary carcinoma: a SEER database analysis. Neurosurg Rev (2014) 37:279–85. doi: 10.1007/s10143-014-0525-y PMC4322934 24526366

[12] Chen Y Wang CD Su ZP Chen YX Cai L Zhuge QC. Natural history of postoperative nonfunctioning pituitary adenomas: a systematic review and meta-analysis. Neuroendocrinology (2012) 96:333–42. doi: 10.1159/000339823 22687984

[13] Raverot G Vasiljevic A Jouanneau E. Prognostic factors of regrowth in nonfunctioning pituitary tumors. Pituitary (2018) 21:176–82. doi: 10.1007/s11102-017-0861-3 29288467

[14] Mercado M Melgar V Salame L Cuenca D. Clinically non-functioning pituitary adenomas: pathogenic, diagnostic and therapeutic aspects. Endocrinol Diabetes Nutr (2017) 64:384–95. doi: 10.1016/j.endinu.2017.05.009 28745610

[15] Binnewies M Roberts EW Kersten K Chan V Fearon DF Merad M. Understanding the tumor immune microenvironment (TIME) for effective therapy. Nat Med (2018) 24:541–50. doi: 10.1038/s41591-018-0014-x PMC5998822 29686425

[16] Chen C Xie L Ren T Huang Y Xu J Guo W. Immunotherapy for osteosarcoma: fundamental mechanism, rationale, and recent breakthroughs. Cancer Lett (2021) 500:1–10. doi: 10.1016/j.canlet.2020.12.024 33359211

[17] Lazarus J Maj T Smith JJ Perusina Lanfranca M Rao A D'Angelica MI. Spatial and phenotypic immune profiling of metastatic colon cancer. JCI Insight (2018) 3:e121932. doi: 10.1172/jci.insight.121932 30429368 PMC6302940

[18] Marques P Barry S Carlsen E Collier D Ronaldson A Awad S. Chemokines modulate the tumour microenvironment in pituitary neuroendocrine tumours. Acta Neuropathol Commun (2019) 7:172. doi: 10.1186/s40478-019-0830-3 31703742 PMC6839241

[19] Principe M Chanal M Ilie MD Ziverec A Vasiljevic A Jouanneau E. Immune landscape of pituitary tumors reveals association between macrophages and gonadotroph tumor invasion. J Clin Endocrinol Metab (2020) 105:dgaa520. doi: 10.1210/clinem/dgaa520 32785693

[20] Zhou W Zhang C Zhang D Peng J Ma S Wang X. Comprehensive analysis of the immunological landscape of pituitary adenomas: implications of immunotherapy for pituitary adenomas. J Neurooncol (2020) 149:473–87. doi: 10.1007/s11060-020-03636-z 33034841

[21] Lupi I Manetti L Caturegli P Menicagli M Cosottini M Iannelli A. Tumor infiltrating lymphocytes but not serum pituitary antibodies are associated with poor clinical outcome after surgery in patients with pituitary adenoma. J Clin Endocrinol Metab (2010) 95:289–96. doi: 10.1210/jc.2009-1583 PMC2805498 19875479

[22] Yagnik G Rutowski MJ Shah SS Aghi MK. Stratifying nonfunctional pituitary adenomas into two groups distinguished by macrophage subtypes. Oncotarget (2019) 10:2212–23. doi: 10.18632/oncotarget.26775 PMC6481336 31040912

[23] Knosp E Steiner E Kitz K Matula C. Pituitary adenomas with invasion of the cavernous sinus space: a magnetic resonance imaging classification compared with surgical findings. Neurosurgery (1993) 33:610–7. doi: 10.1227/00006123-199310000-00008 8232800

[24] Guo J Fang Q Liu Y Xie W Li C Zhang Y. Screening and identification of key microenvironment-related genes in non-functioning pituitary adenoma. Front Genet (2021) 12:627117. doi: 10.3389/fgene.2021.627117 33986766 PMC8110910

[25] Kim D Langmead B Salzberg SL. HISAT: a fast spliced aligner with low memory requirements. Nat Methods (2015) 12:357–60. doi: 10.1038/nmeth.3317 PMC4655817 25751142

[26] Anders S Pyl PT Huber W. HTSeq–a Python framework to work with high-throughput sequencing data. Bioinformatics (2015) 31:166–9. doi: 10.1093/bioinformatics/btu638 PMC4287950 25260700

[27] Yoshihara K Shahmoradgoli M Martinez E Vegesna R Kim H Torres-Garcia W. Inferring tumour purity and stromal and immune cell admixture from expression data. Nat Commun (2013) 4:2612. doi: 10.1038/ncomms3612 24113773 PMC3826632

[28] Miao YR Zhang Q Lei Q Luo M Xie GY Wang H. ImmuCellAI: a unique method for comprehensive T-cell subsets abundance prediction and its application in cancer immunotherapy. Adv Sci (Weinh) (2020) 7:1902880. doi: 10.1002/advs.201902880 32274301 PMC7141005

[29] Asa SL Casar-Borota O Chanson P Delgrange E Earls P Ezzat S. From pituitary adenoma to pituitary neuroendocrine tumor (PitNET): an international pituitary pathology club proposal. Endocr Relat Cancer (2017) 24:C5–8. doi: 10.1530/ERC-17-0004 28264912

[30] Maletkovic J Dabbagh A Zhang D Zahid A Bergsneider M Wang MB. Residual tumor confers a 10-fold increased risk of regrowth in clinically nonfunctioning pituitary tumors. J Endocr Soc (2019) 3:1931–41. doi: 10.1210/js.2019-00163 PMC6777402 31598573

[31] Barry S Carlsen E Marques P Stiles CE Gadaleta E Berney DM. Tumor microenvironment defines the invasive phenotype of AIP-mutation-positive pituitary tumors. Oncogene (2019) 38:5381–95. doi: 10.1038/s41388-019-0779-5 PMC6755983 30867568

[32] Iacovazzo D Chiloiro S Carlsen E Bianchi A Giampietro A Tartaglione T. Tumour-infiltrating cytotoxic T lymphocytes in somatotroph pituitary neuroendocrine tumours. Endocrine (2020) 67:651–8. doi: 10.1007/s12020-019-02145-y PMC7054228 31875303

[33] Marques P Silva AL Lopez-Presa D Faria C Bugalho MJ. The microenvironment of pituitary adenomas: biological, clinical and therapeutical implications. Pituitary (2022) 25:363–82. doi: 10.1007/s11102-022-01211-5 35194709

[34] Suteau V Collin A Menei P Rodien P Rousselet MC Briet C. Expression of programmed death-ligand 1 (PD-L1) in human pituitary neuroendocrine tumor. Cancer Immunol Immunother (2020) 69:2053–61. doi: 10.1007/s00262-020-02611-x PMC11027687 32445029

[35] Huang X Xu J Wu Y Sheng L Li Y Zha B. Alterations in CD8(+) tregs, CD56(+) natural killer cells and IL-10 are associated with invasiveness of nonfunctioning pituitary adenomas (NFPAs). Pathol Oncol Res (2021) 27:598887. doi: 10.3389/pore.2021.598887 34257554 PMC8262195

[36] Bian Y Huang J Zeng Z Yao H Tu J Wang B. Construction of survival-related co-expression modules and identification of potential prognostic biomarkers of osteosarcoma using WGCNA. Ann Transl Med (2022) 10:296. doi: 10.21037/atm-22-399 35434042 PMC9011312

[37] Langfelder P Horvath S. WGCNA: an r package for weighted correlation network analysis. BMC Bioinf (2008) 9:559. doi: 10.1186/1471-2105-9-559 PMC2631488 19114008

[38] Liu K Hu H Jiang H Liu C Zhang H Gong S. Upregulation of secreted phosphoprotein 1 affects malignant progression, prognosis, and resistance to cetuximab via the KRAS/MEK pathway in head and neck cancer. Mol Carcinog (2020) 59:1147–58. doi: 10.1002/mc.23245 32805066

[39] Feng J Wang J Liu Q Li J Zhang Q Zhuang Z. DAPT, a gamma-secretase inhibitor, suppresses tumorigenesis, and progression of growth hormone-producing adenomas by targeting notch signaling. Front Oncol (2019) 9:809. doi: 10.3389/fonc.2019.00809 31508369 PMC6718711

[40] Ducy P Karsenty G. The family of bone morphogenetic proteins. Kidney Int (2000) 57:2207–14. doi: 10.1046/j.1523-1755.2000.00081.x 10844590

[41] Clement JH Sanger J Hoffken K. Expression of bone morphogenetic protein 6 in normal mammary tissue and breast cancer cell lines and its regulation by epidermal growth factor. Int J Cancer (1999) 80:250–6. doi: 10.1002/(SICI)1097-0215(19990118)80:2<250::AID-IJC14>3.0.CO;2-D 9935207

[42] Dai J Keller J Zhang J Lu Y Yao Z Keller ET. Bone morphogenetic protein-6 promotes osteoblastic prostate cancer bone metastases through a dual mechanism. Cancer Res (2005) 65:8274–85. doi: 10.1158/0008-5472.CAN-05-1891 16166304

[43] Hatakeyama S Gao YH Ohara-Nemoto Y Kataoka H Satoh M. Expression of bone morphogenetic proteins of human neoplastic epithelial cells. Biochem Mol Biol Int (1997) 42:497–505. doi: 10.1080/15216549700202901 9247707

[44] Kawabata A Okano K Uchida K Yamaguchi R Hayashi T Tateyama S. Co-Localization of chondromodulin-I (ChM-I) and bone morphogenetic protein-6 (BMP-6) in myoepithelial cells of canine mammary tumors. J Vet Med Sci (2005) 67:1097–102. doi: 10.1292/jvms.67.1097 16327219

[45] Zhang M Yan JD Zhang L Wang Q Lü SJ Zhang J. Activation of bone morphogenetic protein-6 gene transcription in MCF-7 cells by estrogen. Chin Med J (Engl) (2005) 118:1629–36. doi: 10.1016/j.mcm.2008.08.009 16232348

[46] Hong JH Lee GT Lee JH Kwon SJ Park SH Kim SJ. Effect of bone morphogenetic protein-6 on macrophages. Immunology (2009) 128:e442–450. doi: 10.1111/j.1365-2567.2008.02998.x PMC2753950 19191909

[47] Seki N Hattori A Hayashi A Kozuma S Ohira M Hori T. Structure, expression profile and chromosomal location of an isolog of DNA-PKcs interacting protein (KIP) gene. Biochim Biophys Acta (1999) 1444:143–7. doi: 10.1016/S0167-4781(98)00253-X 9931475

[48] Zhu W Jarman KE Lokman NA Neubauer HA Davies LT Gliddon BL. CIB2 negatively regulates oncogenic signaling in ovarian cancer via sphingosine kinase 1. Cancer Res (2017) 77:4823–34. doi: 10.1158/0008-5472.CAN-17-0025 28729416

[49] Wang X Yang Y Cai WQ Lu Y. The relationship of sphingosine kinase 1 with pyroptosis provides a new strategy for tumor therapy. Front Immunol (2020) 11:574990. doi: 10.3389/fimmu.2020.574990 33123153 PMC7566665

[50] Campos B Centner FS Bermejo JL Ali R Dorsch K Wan F. Aberrant expression of retinoic acid signaling molecules influences patient survival in astrocytic gliomas. Am J Pathol (2011) 178:1953–64. doi: 10.1016/j.ajpath.2011.01.051 PMC3081142 21514413

[51] Han J Kioi M Chu WS Kasperbauer JL Strome SE Puri RK. Identification of potential therapeutic targets in human head & neck squamous cell carcinoma. Head Neck Oncol (2009) 1:27. doi: 10.1186/1758-3284-1-27 19602232 PMC2719634

[52] Ogawa R Ishiguro H Kuwabara Y Kimura M Mitsui A Mori Y. Identification of candidate genes involved in the radiosensitivity of esophageal cancer cells by microarray analysis. Dis Esophagus (2008) 21:288–97. doi: 10.1111/j.1442-2050.2007.00759.x 18477249

[53] Pang J Liu WP Liu XP Li LY Fang YQ Sun QP. Profiling protein markers associated with lymph node metastasis in prostate cancer by DIGE-based proteomics analysis. J Proteome Res (2010) 9:216–26. doi: 10.1021/pr900953s 19894759

[54] Chen R Feng C Xu Y. Cyclin-dependent kinase-associated protein Cks2 is associated with bladder cancer progression. J Int Med Res (2011) 39:533–40. doi: 10.1177/147323001103900222 21672358

[55] Barbus S Tews B Karra D Hahn M Radlwimmer B Delhomme N. Differential retinoic acid signaling in tumors of long- and short-term glioblastoma survivors. J Natl Cancer Inst (2011) 103:598–606. doi: 10.1093/jnci/djr036 21346226

[56] Liu J Sun B Guo K Yang Z Zhao Y Gao M. Lipid-related FABP5 activation of tumor-associated monocytes fosters immune privilege *via* PD-L1 expression on treg cells in hepatocellular carcinoma. Cancer Gene Ther (2022) 29:1951–60. doi: 10.1038/s41417-022-00510-0 35902729

[57] Mhawech-Fauceglia P Walia S Yessaian A Machida H Matsuo K Lawrenson K. Overexpression of HOMER2 predicts better outcome in low-grade endometrioid endometrial adenocarcinoma. Pathology (2018) 50:499–503. doi: 10.1016/j.pathol.2018.03.004 29891190 PMC7703685

[58] Heynen GJ Nevedomskaya E Palit S Jagalur Basheer N Lieftink C Schlicker A. Mastermind-like 3 controls proliferation and differentiation in neuroblastoma. Mol Cancer Res (2016) 14:411–22. doi: 10.1158/1541-7786.MCR-15-0291-T 26785999

[59] Onishi H Ichimiya S Yanai K Umebayashi M Nakamura K Yamasaki A. RBPJ and MAML3: potential therapeutic targets for small cell lung cancer. Anticancer Res (2018) 38:4543–7. doi: 10.21873/anticanres.12758 30061220

[60] Aizawa S Fujiwara Y Contu VR Hase K Takahashi M Kikuchi H. Lysosomal putative RNA transporter SIDT2 mediates direct uptake of RNA by lysosomes. Autophagy (2016) 12:565–78. doi: 10.1080/15548627.2016.1145325 PMC4836006 27046251

[61] Hase K Contu VR Kabuta C Sakai R Takahashi M Kataoka N. Cytosolic domain of SIDT2 carries an arginine-rich motif that binds to RNA/DNA and is important for the direct transport of nucleic acids into lysosomes. Autophagy (2020) 16:1974–88. doi: 10.1080/15548627.2020.1712109 PMC7595612 31944164

[62] Yi D Zhang D He J. Long non-coding RNA LIFR-AS1 suppressed the proliferation, angiogenesis, migration and invasion of papillary thyroid cancer cells via the miR-31-5p/SIDT2 axis. Cell Cycle (2021) 20:2619–37. doi: 10.1080/15384101.2021.1995129 PMC8726651 34781815

