# Supplementary material for: Identification of biomarkers associated with the invasion of nonfunctional pituitary neuroendocrine tumors based on the immune microenvironment
Source: Front Endocrinol (Lausanne). 2023 Jul 14;14:1131693. doi: 10.3389/fendo.2023.1131693 (PMC10376796; doi:10.3389/fendo.2023.1131693)
Supplement: Supplementary file 4 [file Table_3.docx]

| **Table S3: Summary of qRT-PCR validated patients** **demographics and characteristics** | | | |
| --- | --- | --- | --- |
| **Characteristic** | | **Group** | |
|  |  | CS invasive  (n) | CS non-invasive (n) |
| **Sex** |  |  |  |
|  | female | 5 | 2 |
|  | male | 3 | 6 |
| **Age** |  |  |  |
|  | ≤ 52 | 6 | 5 |
|  | > 52 | 2 | 3 |
| **Pathological type** |  |  |  |
|  | GAs | 1 | 5 |
|  | SAs | 7 | 3 |
| **Volume classification** | macroadenoma | 2 | 8 |
|  | gaint adenoma | 6 | 0 |

GTs, gonadotroph tumors; SCTs, silent corticotroph tumors; CS, cavernous sinus.
